# The effects of parental education on male mortality: evidence from the first wave of compulsory schooling laws

**DOI:** 10.1007/s00148-025-01134-y

**Published:** 2025-11-12

**Authors:** Hamid Noghanibehambari, Vikesh Amin, Jason Fletcher

**Affiliations:** 1 College of Business, Austin Peay State University, Marion St, Clarksville, TN 37040 USA; 2 Department of Economics, Central Michigan University, 1200 S Franklin St, Mt Pleasant, MI 48859 USA; 3 La Follette School of Public Affairs, University of Wisconsin-Madison, 1225 Observatory Drive, Madison, WI 53706-1211 USA

**Keywords:** Mortality, Longevity, Education, Intergenerational effects, Historical data, I18, I26, J18, H75

## Abstract

This paper investigates the causal impact of fathers’ education on sons’ longevity by linking the full count 1940 US census to Social Security Administration death records and using the first wave of compulsory schooling laws from 1875 to 1912 as instruments for education. OLS estimates suggest small protective effects—conditional on children surviving until age 35, an extra year of fathers’ education increases sons’ age at death by 0.75 months. IV estimates are substantially larger, with an extra year of a father’s education increasing his son’s age at death by 5.6 months. We also find that an extra year of fathers’ education increases sons’ education by 0.22 years, conditional on children surviving till 16 years. This suggests that intergenerational transmission of human capital is a channel linking fathers’ education to children’s longevity. In addition, we find significant improvements in sons’ occupational status associated with higher paternal education, indicating that occupational mobility is another important pathway through which parental education improves long-term health and longevity.

**Supplementary Information:**

The online version contains supplementary material available at 10.1007/s00148-025-01134-y.

## Introduction

Research consistently documents striking education disparities in mortality. While these education-mortality gradients are robust, having been replicated across birth cohorts and countries, the evidence on the causal relationship is unclear. Several studies have used exogenous variation in schooling arising from changes in Compulsory schooling laws (CSLs) to identify the causal relationship. Lleras-Muney (2005) constructed synthetic cohorts using US decennial censuses to compute 10-year mortality rates and used compulsory schooling and child labor laws from 1915 to 1939 as instruments for education. She found large protective effects—an extra year of education reduced 10-year mortality rates by over 6 percentage points. Fletcher (2015) used the NIH/AARP Diet and Health study and found that an extra year of education reduced the likelihood of death over a 10-year period by 6.9 percentage points, though the estimate was imprecise. However, Mazumder (2008) showed that the results in Lleras-Muney (2005) were not robust to the inclusion of state-specific trends. Outside the USA, studies using CSLs have reported null effects for England (Clark & Royer 2013), France (Albouy & Lequien 2009), and Sweden (Meghir et al. 2018), but protective effects have been found in the Netherlands (van Kippersluis et al. 2011) and Romania (Malamud et al. 2021).

Other studies have identified the causal effect of education by comparing mortality and education outcomes within twin pairs. Using male twin pairs from the linked complete-count 1920 and 1940 US Census and death records from the Social Security Administration, Halpern-Manners et al. (2020) found that an extra year of education increased age at death by 0.35 years. Lleras-Muney et al. (2022) linked the complete-count 1940 US Census to death records from Family Tree and found a slightly larger effect in twin fixed-effect models—an extra year of education increased age at death by 0.45 years for both men and women. Twin studies for Scandinavia have reached different findings, with null effects for Denmark (Behrman et al. 2011) but protective effects for Sweden (Lundborg et al. 2016).

The evidence on the causal effects of education on mortality has focused exclusively on educational effects within the same generation (i.e., those directly affected). Little is known about whether there is a protective causal intergenerational effect of education on the mortality/longevity of the next generation. Studies have documented that higher parental education is associated with lower adult mortality risk (Huebener 2019; Lee & Ryff 2019; Montez & Hayward 2011). There are several possible pathways linking parental education to adult mortality risk. Parental education can influence adult mortality risk directly through biological imprinting processes. Lower parental education is associated with higher probabilities of low birth weight (Chevalier & O’Sullivan 2007; Chou et al. 2010; Noghanibehambari et al. 2022), which is a marker of inadequate nutrition and, in turn, is linked to higher risks of hypertension (Falkner 2002), stroke (Lawlor et al. 2005), and cardiovascular disease (Liang et al. 2021) in adulthood. More-educated parents are more likely to have better access to health and medical care for their children. Parental education can also shape adult circumstances that directly affect health. For instance, more-educated parents are more likely to live in affluent neighborhoods, and their children are more likely to attend higher-quality schools where peers are less likely to engage in risky health behaviors, such as smoking. In turn, these children are more likely to achieve higher educational attainment themselves, which is associated with better health outcomes and lower mortality.

Though the link between parental education and adult mortality risk is well established, it remains unclear whether the relationship is causal for at least two reasons. First, observed associations are likely confounded by other unobserved factors correlated with parental education and adult health/mortality. More-educated parents are likely to send their children to higher-quality schools, but the choice of schools is not random. Parents who care strongly about education may elect to live in “good” neighborhoods with high-quality schools and invest more in their children’s education and health. More-educated parents may have children with higher “innate” ability, which is correlated with adult education and health/mortality. Second, a large literature in economics has estimated the causal effects of parental education on children’s health and education and found mixed results. In an influential study, Currie and and Moretti (2003) used the availability of colleges in women’s county of residence at age 17 as an instrument for maternal education. They found that an additional year of college education for mothers reduced the risk of low birth weight by 1 percentage point. Other studies using variation in education from changes in CSLs have found no causal effects of parental education on child health outcomes from birth to age 16 (Arendt et al. 2021; Carneiro et al. 2013; Lindeboom et al. 2009; Silles 2015). Reviewing the literature on causal effects of parental education on children’s education, Holmlund et al. (2011) conclude that intergenerational schooling associations are largely driven by selection and that the causal effect is small at best. The inconclusive evidence on the causal effects of parental education on children’s health and education sheds further doubt on the causal relationship between parental education and adult mortality, as these are two important pathways through which parental education affects adult mortality.

To our best knowledge, only Noghanibehambari and and Fletcher (2024a, b) have attempted to identify the causal effect of paternal education on longevity. They constructed a longitudinal sample of 132,810 sibling fathers and their children based on the US 1940 full count census and Social Security Administration death records and employed cousin fixed-effect models. This approach relates the age at death of cousins to their (sibling) fathers’ education, thereby controlling for a share of the unobserved shared family and genetic factors. Results from cousin fixed-effect models were similar to OLS estimates and showed that conditional on child survival till age 47, the age at death of children whose fathers have a high school (college) education was 2.6 (4.6) months higher than those with a father with elementary/no education. Their estimates, though, may still be biased by sibling-specific factors that are not differenced out. For example, genetic factors are not fully controlled for, as siblings only share 50% of their genetic makeup. Parents may engage in discriminatory treatment of their children that is related to child characteristics, which in turn affects children’s education.

**Fig. 1:**
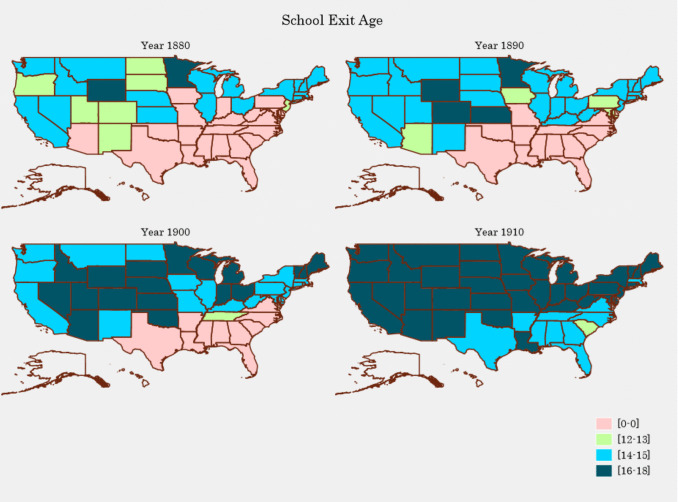
Geographic distribution of school exit age

This paper contributes to the literature by providing new evidence on the causal effect of parental education on adult longevity. We use the same data sources as Noghanibehambari and Fletcher (2024a, b), but use variation in schooling arising from CSLs for identification. Employing CSLs to identify causal effects of education in the USA is a common approach, having been used to estimate causal effects of education on wages, health, and crime (Bennett 2018; Lochner 2004; Oreopoulos & Salvanes 2011; Stephens & Yang 2014). These studies employ the second wave of CSLs (between 1915 and 1939) that focused on high school attendance. In contrast, we use the first wave of CSLs between 1875 and 1912 for identification. Massachusetts was the first state to enact a CSL in 1852. The importance of education gained more attention in 1871 when the Republican Party launched a public-school crusade. Gradually, states enacted CSLs, and by 1900, nearly all states except for those in the South had enacted CSLs. All states had CSLs by 1920. Initial laws set the entry and dropout ages as 8 and 14 years, respectively. Over time, entry ages were generally lowered, while exit ages were raised. Figure 1 shows the geographic distribution of school exit ages over time. As can be seen, by 1910, all states had CSLs, and the majority had an exit age between 16 and 18 years.^1^ CSLs were complemented with child labor laws that allowed employed children to stop attending school before the exit age in a CSL (usually after the child had attained a certain level of education). States often required working children to attend continuation school (part-time or evening school) that supplemented their employment. Enforcement of CSLs varied over time but was bolstered after the enumeration of the 1900 Census revealed that 25% of children between the ages of 10–15 were employed. This led to increased public awareness and state-level changes to improve education and limit child labor.

We find small protective effects of fathers’ education based on OLS regressions. Conditional on children surviving until age 35, an extra year of father’s schooling increases a son’s age at death by 0.75 months. Our IV estimates are substantially larger than OLS estimates and show that, conditional on children surviving till 35 years, an extra year of fathers’ education increases sons’ age at death by 5.61 months.^2^ In the presence of heterogeneous treatment effects, IV estimates reflect a Local Average Treatment Effect (LATE) for individuals (“compliers” who increased their education because of the CSLs, i.e., those whose schooling would have been lower in the absence of such laws). We note that our LATE estimates reflect the effect of completing primary schooling, given that the first wave of CSLs focused on education up to age 14.

The LATE interpretation requires the satisfaction of the exclusion restriction assumption that the law changes are orthogonal to other policy and environmental changes that influence children’s longevity and are correlated with parental education. These endogenous influences may produce pre-treatment effects, and compulsory schooling laws could ride on those trends. We empirically test for this using an event-study estimation and find no evidence for this concern. In analyses to explore mechanisms, we find significant increases in children’s educational attainment, suggesting that improvements in human capital are a likely mechanism channel. In addition, we find that higher parental education leads to significantly improved occupational attainment among sons, indicating that occupational mobility may serve as an additional pathway linking parental education to longevity. Therefore, this study also adds to the literature on the intergenerational transmission of human capital by providing evidence based on the first wave of compulsory schooling laws in the USA.

The rest of the paper is organized as follows. We describe the data sources in Section 2 and outline our econometric approach in Section 2.1. The results are presented in Section 2.2. Finally, Section 3 concludes the paper.

## Data and sample selection

### Data sources

The primary source of data is the Social Security Administration’s Death Master Files (DMF) extracted from the CenSoc project (Breen et al. 2023; Goldstein et al. 2021). The DMF data contains death records of male individuals who died between 1975 and 2005. There are two advantages to using the DMF data. First, the DMF data can be linked to the full-count 1940 census, which means we can observe individual and parental characteristics in 1940. Specifically, it allows us to extract information on fathers’ education and fathers’ birth-state. Second, the DMF data contains millions of observations. This contrasts with many alternative data sources with much smaller sample sizes, such as the Health and Retirement Study and the National Longitudinal Mortality Study.

One issue with the DMF-1940-census linked data is that we only observe parental characteristics for those who live in their original households and with their parents. The limited cohort window of observation results in a limited parental cohort window and less cross-cohort variation in schooling law exposure. We address this limitation by linking 1940 records to historical censuses 1900–1930 using cross-census linking rules provided by the Census Linking Project (Abramitzky et al. 2020). We start by linking individuals between 1940 and 1900. If there is parental information in 1900, we use it as the original household’s characteristics. For the rest of the observations, we move to linking 1940 and 1910 and repeat the process.

Although we can deduce parental birthplace and measures of socioeconomic status in 1900–1930, we do not have information on education as the 1940 census is the first census to report education. Therefore, we again employ cross-census linking to link parents back to their 1940 census records. We should note that they may or may not be observed in the same household as their children during the latter linking. This is not an issue as we already know the location of their children in 1940 and the household record number in 1900–1930 censuses for both parents and children. In fact, the sole purpose of linking parents from 1900–1930 to 1940 is to extract the education information. Linking techniques are reliable only for male individuals, since females usually change their surnames after marriage; parental linking is possible only for fathers. For mothers, we only use the available information in the 1940 census.

The cross-census linking allows us to add more observations to the final sample by having more individuals with parental characteristics. However, there is another advantage of searching for individuals in historical censuses. The full-count historical censuses report county-of-residence at the time of the census. By observing individuals during their childhood years, we can infer their county-of-birth by proxying the county of census observation. We use the county-of-residence in the first historical census in which an individual appears as a proxy for their county of childhood. If the individual is not linkable to historical censuses, we use the county-of-residence in 1940 as the proxy. This information on place-of-birth is important given the growing evidence pointing to the relevance and influence of local area conditions during early life on lifecycle outcomes and specifically old-age mortality and longevity (Aizer et al. 2016; Lindeboom et al. 2010; Noghanibehambari et al. 2024; Noghanibehambari & Engelman 2022; Schmitz & Duque 2022; Van Den Berg et al. 2006, 2009).

The complexity of linking data across historical censuses to the 1940 census, as well as the 1940 census to the Social Security death records, brings concerns that the final selected sample is different from the original population in a way that confounds our estimates.^3^ For instance, if individuals in states with stricter compulsory schooling laws are more likely to appear in the final sample than in other states, the coefficients may overstate the effects by inflating through overrepresentation of the treated population. We empirically test this concern by investigating the association between being in the final sample from the original 1940 population and parental exposure to state-level educational laws. In so doing, we focus on individuals born between 1900 and 1940 observed in the full count 1940 census. We implement similar sample selections and merge this data with our final sample of this study. We generate a dummy variable that indicates successful merging. We then regress this indicator on parental birth state educational laws, conditional on fixed effects and covariates (similar to the identification strategy of Section 2.1). These results are reported in Appendix Table A-3. For both samples of mothers and fathers, we observe quite small and mostly insignificant associations between state-level educational laws and the successful merging indicator. These results reduce the concern of endogenous data linking.^4^

Data on CSLs and child labor laws comes from Clay et al. (2021). Their dataset builds on prior work by Lleras-Muney (2002) and Goldin and and Katz (2011) by extending the previous coding to 1880. They also follow Stephens and and Yang (2014) and use an iterative process to calculate years of required schooling for each state-year birth cohort.^5^

### Sample selection

The original DMF-census data contains approximately 7.8 million records. About 30 % of observations either have parental information (parents are alive and children are living with their parents) or we can extract their parental information from historical censuses. Hence, the final sample contains 1,913,593 observations. Summary statistics of the final sample are reported in Table 1. We should highlight that the sample covers male individuals only. The average age at death in the final sample is 814.1 months (67.8 years). The sample covers cohorts who died between 1975 and 2005 and were born between 1885 and 1940 to parents born between 1882 and 1915. About 7 % of observations are nonwhite, and 1 % are Hispanic. The average father’s years of schooling is 7.99 years. Fathers who were born in states with 1–5, 6, 7, 8, and 9–10 years of compulsory schooling law account for about 11, 25, 16, 12, and 1 % of observations, respectively.^6^

**Table 1:** Summary statistics

	Mean	SD	Min	Max
Death age (months)	814.189	118.226	418.000	1231.000
Birth year	1925.779	6.710	1900.000	1940.000
Death year	1993.631	8.428	1975.000	2005.000
Nonwhite	0.066	0.248	0.000	1.000
Hispanic	0.011	0.105	0.000	1.000
Father’s birth year	1896.363	7.816	1882.000	1915.000
Father’s years schooling	7.997	3.327	0.000	23.000
Father’s years schooling $>4$	0.863	0.344	0.000	1.000
Father’s years schooling > 7	0.628	0.483	0.000	1.000
Father’s birth-state compulsory schooling 1–5 years	0.110	0.314	0.000	1.000
Father’s birth-state compulsory schooling 6 years	0.251	0.434	0.000	1.000
Father’s birth-state compulsory schooling 7 years	0.156	0.363	0.000	1.000
Father’s birth-state compulsory schooling 8 years	0.123	0.329	0.000	1.000
Father’s birth-state compulsory schooling 9–10 years	0.009	0.096	0.000	1.000
Mother’s birth year	1899.922	7.727	1882.000	1915.000
Mother’s years schooling	8.356	2.969	0.000	20.000
Mother’s years schooling $>4$	0.911	0.285	0.000	1.000
Mother’s years schooling > 7	0.678	0.467	0.000	1.000
Mother’s birth-state compulsory schooling 1–5 years	0.114	0.318	0.000	1.000
Mother’s birth-state compulsory schooling 6 years	0.210	0.407	0.000	1.000
Mother’s birth-state compulsory schooling 7 years	0.162	0.368	0.000	1.000
Mother’s birth-state compulsory schooling 8 years	0.169	0.375	0.000	1.000
Mother’s birth-state compulsory schooling 9–10 years	0.062	0.242	0.000	1.000
Schooling/(age-5) (conditional on age $\ge$ 7)	2.156	3.436	0.000	20.000
Own schooling (conditional on age $\ge$ 16)	7.070	3.878	0.000	20.000
Own socioeconomic index (conditional on age > 16)	22.537	17.275	3.000	96.000
Observations	1,913,593			

## Econometric method

### Econometric approach

Our main specification for estimating the effect of parental education relates the age at death of child $$i$$ born in cohort $$b$$ and in county $$c$$ whose parent is born in state $$s$$ in census region $$r$$ in year $$y$$ $${(D}_{ibcsry}$$) to parental education ($${PE}_{sry})$$, a vector of individual characteristics ($${X}_{i})$$ that includes race and ethnicity, birth cohort fixed effects for the child $$({\gamma }_{b})$$, county-of-birth fixed effects for the child ($${\xi }_{c}),$$ parental state-of-birth fixed effects ($${\eta }_{s}$$), parental region-of-birth-by-birth-cohort fixed effects ($${\gamma }_{ry})$$, and an error term $${(u}_{ibcsry})$$:

1 $$D_{ibcsry}=\beta_0+\beta_1{PE}_{sry}+X_i'\theta+\gamma_b+\xi_c+\eta_s+\gamma_{ry}+u_{ibcsry}$$

OLS estimates though are likely biased from unobserved factors correlated with parental education and children’s longevity. We identify the causal effect by using variation used in CSLs to predict parental educational attainment by estimating the first-stage equation:

2 $${PE}_{sry}=\alpha_0+\alpha_1{LawYears}_{sry}+X_i'\theta+\gamma_b+\xi_c+\eta_s+\gamma_{ry}+u_{ibcsry}$$

where $LawYears$ is a set of dummy variables for requiring (1) 1–5 years of schooling, (2) 6 years of schooling, (3) 7 years of schooling, (4) 8 years of schooling, and (5) 9 years of schooling or more. Dummy variables are used for $LawYears$ because the effect of requiring one more year of schooling may not be linear throughout the distribution of required schooling. A key assumption to identify causal effects of education in this framework is that all other changes which occur across states and regions are uncorrelated with law changes, educational improvement, and the outcome (longevity in our case). We account for this through the inclusion of parental-state-of- birth fixed effects and region-by-year-of-parental-birth-cohort fixed effects. These fixed effects ensure that effects are identified by changes *within* states and region over time and that states are being compared to other states in their census region for the same birth cohorts.^7^ We also present evidence from even-study analysis in the next section to support this identification assumption.

## Results

### First-stage results

First-stage estimates of the effect of compulsory schooling laws on parental education are shown in Table 2. All regressions control for the child’s birth-county fixed effects and birth year fixed effects, parental birth-state fixed effects, and parental region-of-birth-by-birth-cohort fixed effects. Column 1 shows that compared to states with no compulsory schooling, those who were born in states with compulsory schooling laws have significantly higher educational attainment. Moreover, there is a monotonic pattern in marginal effects, suggesting increases in the effects as the required years of education increase. For instance, those born in states with 1–5 required years of schooling have 0.01 additional years of schooling. Those born in states with 6 years of required schooling have an additional 0.10 years of schooling, and those born in states with 9–10 years of required schooling have 0.19 additional years of schooling.

**Table 2:** Effect of compulsory schooling laws on fathers’ education

	***Outcomes:***		
	Years of schooling	Years of schooling > 4	Years of schooling > 7
	(1)	(2)	(3)
Law requires 1–5 years	0.009	0.001	0.002
	(0.018)	(0.002)	(0.003)
Law requires 6 years	0.096***	0.013***	0.016***
	(0.020)	(0.002)	(0.003)
Law requires 7 years	0.087***	0.011***	0.016***
	(0.023)	(0.003)	(0.004)
Law requires 8 years	0.209***	0.015***	0.036***
	(0.028)	(0.003)	(0.005)
Law requires 9–10 years	0.186***	0.001	0.026***
	(0.039)	(0.005)	(0.006)
Mean DV	7.997	0.863	0.628
Observations	1,913,592	1,913,592	1,913,592

Standard errors, clustered on father’s birth-state-birth-year are in parentheses. Regressions include child’s birth-county fixed effects, child’s birth year fixed effects, parental birth-state fixed effects, and parental region-of-birth-by-birth-cohort fixed effects. Regressions also include race/ethnicity dummies as individual covariates

^*^p < 0.1; **p < 0.05; ***p < 0.01

In columns 2 and 3, we explore the effects on two binary outcomes indicating fathers’ education is more than 4 and 7 years, respectively. These thresholds point to finishing elementary and middle school education, though they are still arbitrary cut-off points as they do not account for grade repetitions. The compulsory schooling laws had positive and significant effects on both outcomes. Laws requiring 7, 8, and 9–10 years of schooling also had much larger effects on the likelihood of having more than 7 years of schooling. For example, laws requiring 7 (8) years of schooling increased the probability of having more than 7 years of schooling by 1.6 (3.6) percentage points. In comparison, compulsory schooling laws for 7 (8) years only increased the probability of having more than 4 years of schooling by 1.1 (1.5) percentage points. This is what one would expect from laws that were enforced.

**Fig. 2:**
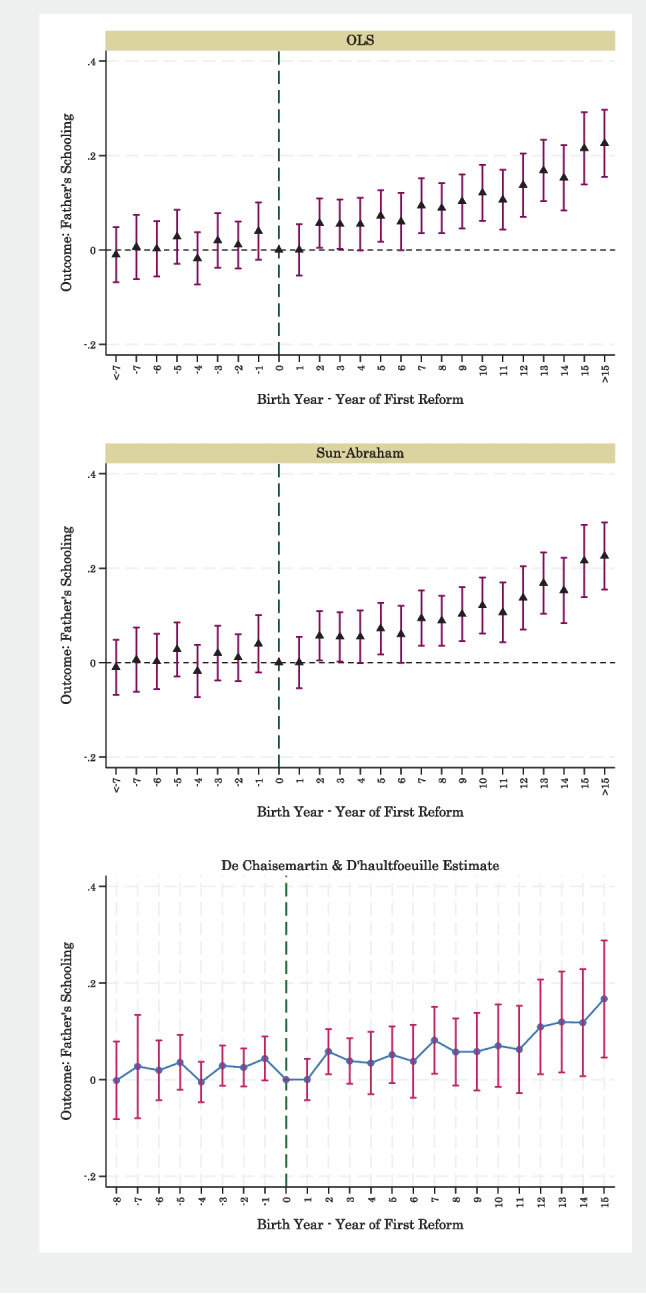
Event study analysis of compulsory schooling laws

An important concern is that compulsory schooling laws were enacted during a period of rising educational attainment and may not have been the cause of those increases. Figure 2 shows results from an event-study analysis to examine this concern. The event-time coefficients from OLS regressions are small, often negative, and indistinguishable from 0 before the establishment of compulsory schooling laws. The coefficients start to rise and become statistically significant 6 years after the laws were implemented. A similar pattern is observed in the middle panel of Fig. 2, which shows results from the Sun and Abraham (2021) specification for event-study analysis. The bottom panel of this figure shows the results of De Chaisemartin and d’Haultfoeuille (2024) and suggests a very similar pattern in coefficients to those reported for OLS estimates. Overall, Fig. 2 shows that educational attainment was not increasing prior to the implementation of compulsory schooling laws.^8^

### Main results

Our main results are given in Table 3.^9^ The OLS estimate in column 1 shows that, conditional on surviving to age 35 years, an extra year of fathers’ education is associated with a 0.75-month increase in children’s (sons’ as the sample covers male individuals only) age at death. This intergenerational OLS association is much smaller than OLS associations between education and longevity within the same generation. For example, OLS estimates in Halpern-Manners et al. (2020) and Lleras-Muney et al. (2022) from the 1940 full count US census indicate one extra year of education is associated with about 0.40 more years (or 4.8 months) of life. In contrast, the IV estimate in column 2 is over 7.5 times larger—an extra year of fathers’ education increases children’s longevity by 5.6 months. The discrepancy between the OLS and IV estimates is likely because they represent different treatment effects. OLS estimates represent average treatment effects (ATE), whereas in the presence of heterogeneous treatment effects, IV methods identify a LATE. Specifically, the IV estimates reflect the average effect of completing primary schooling for fathers (who increased their education because of the CSLs) on children’s longevity. Columns 3–4 and 5–6 show results for white and nonwhite individuals, respectively. The protective effect of fathers’ education is predominantly concentrated on nonwhite individuals. Based on IV estimates, an extra year of fathers’ education increases the longevity of white and nonwhite children by 5.1 and 9.4 months, respectively. The IV estimate for nonwhite individuals, though, is biased by weak IV, as indicated by the low first-stage *F*-statist ic.^10^

**Table 3:** Effect of fathers’ education on sons’ longevity

	*Outcome: age-at-death (months)/sample/method:*					
	Full		Whites		Nonwhites	
	OLS	IV	OLS	IV	OLS	IV
	(1)	(2)	(3)	(4)	(5)	(6)
Father’s years of schooling	0.745***	5.609**	0.657***	5.139**	0.523***	9.352*
	(0.025)	(2.198)	(0.026)	(2.617)	(0.102)	(5.539)
Mean DV	814.189	814.189	816.005	816.005	788.306	788.306
SD DV	118.225	118.225	117.893	117.893	119.911	119.911
Observations	1,913,592	1,913,593	1,787,864	1,787,866	125,474	125,727
First-stage *F*-statistic		16.521		14.684		6.285

Standard errors, clustered on father’s birth-state-birth-year are in parentheses. Regressions include child’s birth-county fixed effects, child’s birth year fixed effects, parental birth-state fixed effects, and parental region-of-birth-by-birth-cohort fixed effects. Regressions also include race/ethnicity dummies as individual covariates

*DV* dependent variable; *SD* standard deviation

^*^p < 0.1; **p < 0.05; ***p < 0.01

As noted in Sect. 2, we focus on fathers’ education because linking techniques are not reliable for women.^11^ Nevertheless, results for women are presented in Appendix Table A-4 and Appendix Table A-9. The conclusions are similar to those found for fathers’ education. IV estimates uncover large proactive effects of mothers. An additional year of maternal education increases children’s longevity by 9.92 months, conditional on surviving till age 35. This is substantially larger than the OLS estimate of 0.77 months. Similar to fathers’ education, the effect of mothers’ education is concentrated among nonwhite individuals. An extra year of mother’s education increases children’s longevity by 8.67 months for white individuals and 14.97 months for nonwhite individuals.^12^

Our IV estimates for fathers’ education are much larger than the sibling-based estimates reported in Noghanibehambari and Fletcher (2024a, b). They found that, conditional on child survival till age 47, an additional year of fathers’ education is associated with 0.4 months higher longevity. We also note that our IV intergenerational effects for fathers’ and mothers’ education are comparable in some instances to other “causal” estimates of education and longevity within the same generation. For example, family fixed-effect estimates in Halpern-Manners et al. (2020) and Lleras-Muney et al. (2022) indicate that an extra year of education is associated with a 4.8 month increase in age at death. Fletcher & and Noghanibehambari (2023) explore the effects of college openings on college education and mortality. They estimate a treatment-on-treated effect of college education on longevity of about 1 additional year. Therefore, the effect of college education is equivalent to about 2 years of additional fathers’ education.

### Robustness checks

An important concern is that the treated groups may differ in both observable and unobservable ways, including baseline health and longevity, and that the results may partly reflect these pre-existing differences. In Appendix Table A-6, we explore the association between race and ethnicity (as predetermined individual characteristics) on parental state-level educational policies. We observe that whites and non-Hispanics are more exposed to stricter educational policies. However, compared to the outcome mean, the observed associations are quite small in magnitude. For instance, exposure to compulsory schooling laws of 8 and 9–10 years is associated with a 1.4 and 1.5 % higher likelihood of being white. The coefficient on white in column 2 of Table 3 (i.e., the white-nonwhite difference in longevity conditional on covariates) is 3.6 (se = 1.2). Therefore, the potential endogenous change in the sample’s racial composition can explain most 0.05 months of longevity, which is only 0.8% of the effects we find in Table 3.

Further, in Appendix Table A-8, we construct a state-by-year panel (1880–1920) and examine the association between educational laws and other policies and state characteristics. We do not find a consistent association between these policies and the share of dry counties in the state, birth registration laws, suffrage laws, and poll tax laws.^13^ However, we observe a negative association between these policies and measures of socioeconomic index (column 5) and the share of literate people (column 7). The states that passed stricter policies are those with a higher male labor force participation rate (column 6).

To complement the analysis of this section, we implement two robustness checks. First, we interact birth state fixed effects and birth year fixed effects with dummies for race and ethnicity to allow for flexibility of place and time effects across different racial groups. The results are reported in Appendix Table A-11. We observe a set of coefficients that are very similar in magnitude to the main results. Second, we include several time-varying state covariates, including socioeconomic index, literacy rate, labor force participation rate, share of married people, share of different race groups, and share of different age groups. These results are reported in Appendix Table A-12. For the full sample and the sample of white people, we observe comparable coefficients to the main results. However, the coefficient on nonwhites is substantially larger than those reported in the paper, although very small first-stage F-statistics make this estimate unreliable.

In Appendix Table A-14, we examine the robustness of the results to including fathers’ birth-state by birth-year linear trend. We observe a slight increase in the magnitude of the IV estimate, although it is now stylistically insignificant, limiting further interpretations.

In Appendix Table A-15, we further examine the robustness of our results by introducing additional controls related to early-life conditions in fathers’ birth states. Prior research documents long-term mortality effects from exposure to historical disease environments, particularly the 1918 influenza pandemic and the 1916 polio epidemic (Almond 2006; J. M. Fletcher 2018c, 2018b, 2018a; Mazumder et al. 2010; Meyers & Thomasson 2021; Noghanibehambari & Fletcher 2023c, 2024b). Other studies highlight both the detrimental effects of exposure to malaria and hookworm and the health gains from early 20th-century eradication campaigns (Bleakley 2007; Bleakley & Lange 2009; Venkataramani 2012). In columns 2–6 of Appendix Table A-15, we include controls for these disease exposures and find that the estimated coefficients become even larger in magnitude.^14^

In columns 7–9, we adjust for differences in school quality across states by including controls for school attendance rates, average school year length, and term length. These specifications also yield larger coefficient estimates, suggesting our main results are not driven by variation in school infrastructure.

Finally, given evidence linking early-life exposure to the prohibition movement and to birth registration laws with improved long-term health (Fagernäs, 2014; Nilsson 2017; Noghanibehambari & Fletcher 2023b, 2023a; Weinberg et al. 2008), we include controls for these policy environments in Columns 10–11. We find that the estimated effects of fathers’ education remain virtually unchanged, reinforcing the robustness of our findings.

## Nonlinearities in the effects of fathers’ education

Given that compulsory schooling laws tended to induce discrete jumps in educational attainment, we explore whether the longevity benefits of paternal education are nonlinear and concentrated around specific thresholds. Specifically, we replace the continuous measure of paternal schooling with a series of binary indicators for exceeding discrete schooling thresholds (e.g., > 4, > 5, … > 12 years). These results, reported in Appendix Table A-17, reveal an inverted U-shaped pattern in the estimated effects.

The largest gains in sons’ longevity are observed for thresholds at 5 to 7 years of paternal schooling, with significant increases in age at death ranging from approximately 34 to 74 months. The magnitude of the effect declines substantially at higher levels of paternal education, and coefficients become statistically insignificant beyond the 8-year threshold. This pattern suggests that the longevity benefits for children are strongest when fathers move from low to moderate levels of education, particularly around the compulsory schooling thresholds targeted by early CSLs. These findings suggest that the primary longevity benefits for the next generation stem from ensuring basic educational attainment, reinforcing the interpretation of our IV estimates as Local Average Treatment Effects (LATE) for individuals induced into primary schooling by early CSLs.

## Potential mechanism

In this section, we explore the intergenerational transmission of human capital as a mechanism linking fathers’ education to children’s longevity.

One issue is that most of the children in the 1940 census had not completed their education. One way to account for these limitations is to construct a measure that captures the educational level of each child in comparison with other children of the same age. We assume that school age starts at age 6, and each child in each year after that age can attain a maximum of one year of education (and a minimum of zero). Therefore, the ratio of actual attainment reveals a relative measure of education for age, a proxy for progression in the child’s education. Formally, we define this variable as follows: years of education/(age − 6). With this outcome, an additional year of fathers’ education increases children’s expected education by 0.22, which is considerably larger than the OLS estimate of 0.08 (columns 1–2, Table 4).

**Table 4:** Effect of fathers’ education on sons’ education and socioeconomic status (ses)

	*Outcome/method:*					
	Schooling/(age-6) conditional on age $\ge$ 6	Schooling/(age-6) conditional on age $\ge$ 6	Schooling conditional on age $\ge$ 16	Schooling conditional on age $\ge$ 16	SES score conditional on age > 17	SES score conditional on age > 17
	OLS	IV	OLS	IV	OLS	IV
	(1)	(2)	(3)	(4)	(5)	(6)
Father’s years of schooling	0.080***	0.222***	0.325***	0.877***	1.397***	3.272***
	(0.002)	(0.050)	(0.004)	(0.156)	(0.013)	(1.032)
Mean DV	2.156	2.156	9.764	9.764	22.713	22.713
Observations	1,555,172	1,555,172	837,776	837,776	448,726	448,726
First-stage *F*-statistic		12.595		6.167		4.727

Standard errors, clustered on father’s birth-state-birth-year are in parentheses. Regressions include child’s birth-county fixed effects, child’s birth year fixed effects, parental birth-state fixed effects, and parental region-of-birth-by-birth-cohort fixed effects. Regressions also include race/ethnicity dummies as individual covariates

^*^p < 0.1; **p < 0.05; ***p < 0.01

Nevertheless, these are the first IV-based estimates of the intergenerational transmission of human capital in the USA and are similar to existing estimates from other research designs.^15^ Using data on twin fathers and their children from the Minnesota Twin Registry, Behrman & Rosenzweig (2002) found that an extra year of fathers’ education increased children’s education by 0.36 years, and there was no effect of mothers’ education.^16^ Using data on parents and their adopted children from the Wisconsin Longitudinal Study, Plug (2004) found that an additional year of either parent's education increased children’s education by about 0.27 years. Using adopted children and parents from the National Longitudinal Study of Youth, Sacerdote (2004) found that an extra year of fathers’ (mothers’) education increased children’s education by 0.16 (0.22) years. We should note two facts in comparing our results with these studies. First, our IV results use a treatment effect that relies on changes in schooling policies that resulted in the take-up of primary and secondary schooling. This is in contrast with many studies that examine compulsory schooling that enforces high school education and completion. Second, considering the relatively weak first-stage effects and the focus of laws in an environment with fewer available schools and educational resources, the intergenerational impacts reported in column 2 of Table 4 are relatively large compared with other studies.

Alternatively, we can restrict the sample to children above age 16, those who are more likely to have completed their education. In the final sample and for those children with at least 16 years old, about 97 % of fathers were born prior to 1900. Therefore, the caveat of this analysis is that we have much limited variation in exposure to schooling laws specifically for those of the early twentieth century. The OLS estimate in Table 4, column 3, shows that one additional year of fathers’ education is associated with a 0.33-year increase in children’s education. The IV estimate shows an increase of 0.22 years in children’s education, conditional on survival up to age 16, although small first stage *F*-statistics limits further interpretation.,^17^,^18^

As another measure, columns 5 and 6 use the Duncan socioeconomic index (SEI) of children as the outcome. The Duncan SEI is a measure of occupational status based upon the income level and educational attainment associated with each occupation in 1960. The IV estimate shows that an extra year of fathers’ education increases the Duncan SEI by 3 units, which represents a 14% effect relative to the mean SEI of 22.71.

In Appendix Table A-19, we examine the intergenerational effects of fathers’ education on place attainment and occupational mobility. When using average years of schooling in the individual's county of residence in 1940 as an outcome, we find no statistically significant effect, suggesting that paternal education did not substantially influence geographic sorting or place-based human capital accumulation later in life. In contrast, we observe strong and statistically significant effects on multiple measures of occupational status. Specifically, fathers’ education is associated with higher occupational income and earnings scores, as well as increased occupational prestige.^19^ These results suggest that occupational mobility may be an important pathway through which parental education improves long-term health and longevity. Enhanced occupational attainment likely reflects both better labor market opportunities and working conditions, which may contribute to the observed gains in sons’ longevity.

## Conclusion

A growing and relatively large literature evaluates the spillover effects of education across a wide array of areas and outcomes, including crime, civic engagement, political participation, smoking, drinking, and health (Currie & Moretti 2003; Dee 2004; Lochner & Moretti 2004; Tenn et al. 2010). A narrow strand of this research evaluates the intergenerational impacts of education on mortality and examines the role of parental education on children’s mortality outcomes during adulthood and old age (Huebener 2019; Noghanibehambari & Fletcher 2024a). This paper adds to this literature by evaluating the impact of the first wave of CSLs on parental education and children’s old-age longevity during the late nineteenth century, when there were minimal to zero schooling policies in the USA. The introduction of compulsory schooling policies was among the first policy-driven attempts to promote education. Since the late 19th and early twentieth century USA represents the developmental stages of many developing countries today, the results of this paper shed light on the relevance of schooling laws and their externalities for the next generation’s health outcomes.

We employ death records from the Social Security Administration linked with the 1940 full-count census. This linked data provides a unique setting to extract information on parental characteristics as well as individuals’ early-life and late-life outcomes. In addition, the unprecedented large sample size makes the data a distinct source to explore the research question.

Our IV results using compulsory schooling laws as the exogenous instrument suggest approximately 5.6 (9.92) months increase in children’s longevity for one additional year of fathers’ (mothers’) schooling. These estimates are substantially larger than the OLS effects, which imply about 0.75 (0.76) months rise in longevity for an additional year of fathers’ (mothers’) education. Further analyses suggest that improvements in family socioeconomic status, as well as increases in children’s educational attainment and occupational status, are likely mechanisms linking parental education to longevity. The nontrivial effects of education on the next generation’s longevity add to the benefits and positive externalities of policies that aim at promoting educational outcomes, specifically in an environment with limited established educational policies.

## Supplementary Information

Below is the link to the electronic supplementary material.

ESM 1 (PDF 1.01 MB)

## Data Availability

The code and data to replicate the results are available at https://data.mendeley.com/datasets/bmbgt5x42h/1.

## Footnotes

1 In Appendix Figure A-1, we show the geographic distribution of school exit age, the minimum age for school leaving, across states and several years throughout the sample period.

2 We focus on fathers’ education because the linking techniques used are reliable only for men, as women usually change their names after marriage. We nevertheless present results for mothers’ education in the appendix and discuss these in Sect. 2.2.

3 In Appendix Table A-1, we compare several statistics of the final sample with the original population. Specifically, we compare distributions of birth cohort, race, paternal education, and paternal exposure to compulsory schooling laws. We observe some modest differences in several measures, such as father’s education, son’s education, and birth cohort. The final sample covers a lower share of nonwhites and a higher share of families with higher socioeconomic status. These differences have been previously documented in the literature regarding cross census linking as well as linking census with the Social Security death records (Abramitzky et al. 2020; Breen & Osborne 2022). In addition, to account for potential selection on observables, we implement a Heckman two-step selection model (Appendix Table A-16), where the first-stage estimates the probability of inclusion based on early-life characteristics. The second-stage results show that our main estimates are robust to this correction, indicating that selection into the analysis sample is unlikely to be driving our findings.

4 Additionally, in the Appendix Table A-16, we implement the two-step selection correction procedure proposed by Heckman (1979) to address potential concerns about sample selection bias due to data linking. Specifically, we use the original 1940 census population and regress a successful merging indicator on observable characteristics and the full set of fixed effects. From this model, we compute the inverse Mills ratio (IMR), capturing the probability of being included in the final sample conditional on observables. We then include the IMR as an additional control in our main regression specifications. The resulting estimates remain highly consistent with our baseline results, further confirming the robustness of our findings to potential selection concerns.

5 We refer readers to the data appendix in Clay et al. (2021) for specific procedure to calculate required years of schooling.

6 In Appendix Table A-2, we show the brief summary statistics of selected variables across states with different schooling policies.

7 Stephens and Yang (2014) showed that the return to schooling IV estimates for white men based on the 2nd wave of compulsory schooling laws become statistically insignificant and, in some cases, negative when including region by year of birth effects in regressions to control for differential changes across states such as improvements in school quality. Their results highlight the importance of controlling for region by year birth effects.

8 In the top panel of Appendix Figure A-2, we show that the results are robust when we employ Callaway & Sant’Anna (2021)’s estimation method. The bottom panel shows similar results when we de-trend the outcome for pretreatment periods as suggested by Goodman-Bacon (2021).

9 The CenSoc project also provides access to the Numident data, which includes mortality records for both genders. However, its primary limitation relative to the DMF data is the narrower death window—covering only the years 1988–2005. As shown in Appendix Table A-13, OLS estimates become substantially smaller and IV estimates lose statistical significance when using the Numident data. These findings underscore the importance of including earlier death years and suggest that the truncation in the DMF data may, if anything, lead to conservative estimates of the true effects.

10 To help interpret the relatively large IV estimates compared to the OLS results, we explore potential treatment effect heterogeneity that may underlie this discrepancy. In the Appendix Table A-5, we further explore the heterogeneity of our results by stratifying the sample based on fathers’ education, socioeconomic status, and birth region. In columns 1–2 of Appendix Table A-5, we find that the effect of fathers’ education on sons’ longevity is significantly larger among fathers with lower educational attainment (i.e., less than 12 years of schooling). In contrast, columns 3–4 show slightly larger coefficients for the subsample of fathers with socioeconomic status above the median, suggesting that intergenerational benefits may be more pronounced among higher-status families. When we examine heterogeneity by fathers’ birth region in columns 5–8, we find statistically insignificant estimates, likely due to limited power in these subsamples. Nonetheless, the point estimate for fathers born in the Western region is notably larger than those from other regions.

11 The Census Linking Project (as described in Abramitzky et al. (2020)) primarily focuses on linking males across historical US censuses, largely because name-based linking algorithms are more accurate for men due to women's surname changes after marriage. Therefore, cross-census links for women are excluded from core data.

12 The first stage for nonwhite individuals is weak with a *F*-statistic of 7.44. There is no problem of weak instruments for white individuals or in the full sample. First stage results are given in Appendix Table A-9.

13 Several studies point to the influence of these policies on long-term health and mortality (Noghanibehambari & Fletcher 2023b, 2023a; Noghanibehambari & Noghani 2023).

14 The 1890 state-level infant mortality rate is taken from Bleakley (2010). State-level prevalence of influenza and polio are extracted from the Tycho project (Tycho 2021) and interacted with parental birth cohort fixed effects to capture temporal variation. Specifically, we use influenza prevalence from historical surveillance records and polio prevalence from 1916. Additionally, we incorporate state-level malaria risk and hookworm rates for 1910, also from Bleakley (2010), each interacted with parental birth cohort fixed effects.

15 The only other IV paper to use compulsory schooling laws to estimate the intergenerational transmission of human capital is (Oreopoulos et al. 2006). They examine grade repetition and use compulsory schooling laws between 1915 and 1969 as instruments. They found an extra year of fathers’ education reduced the probability that a child repeats a grade by 2–4 percentage points.

16 Antonovics and Goldberger (2005) argue that the results from Behrman and Rosenzweig (2002) are not robust to alternative coding schemes for education and alternative sample selection criteria. Behrman & Rosenzweig (2005) contest Antonovics and Goldberger’s recoding and show that their results are robust to an independent coding scheme.

17 Appendix Table A-9 shows results for mothers’ education. OLS estimates show that one additional year of mothers’ education is associated with a 0.09-year increase in children’s education per age and 0.4-year increase in schooling conditional on age at least 16, whereas the IV estimates are 0.19 and 1.2, respectively.

18 Antonovics and Goldberger (2005) argue that the results from Behrman and Rosenzweig (2002) are not robust to alternative coding schemes for education and alternative sample selection criteria. Behrman and Rosenzweig (2005) contest Antonovics and Goldberger’s recoding and show that their results are robust to an independent coding scheme.

19 We use four standardized measures of occupational status—income, earnings, education, and prestige—which we briefly describe below (based on (Ruggles et al. 2024)). Occupational Income Score: This variable assigns each occupation the median total income (in hundreds of 1950 dollars) earned by individuals in that occupation in 1950. It reflects the economic returns associated with occupational categories based on historical census data. Occupational Earnings Score: This variable ranks occupations by standardized median earned income, reporting the percentile rank of each occupation relative to others in the 1950 labor force. For example, a score of 64 means the occupation has higher earnings than 64% of all occupations. Occupational Education Score: This measure indicates the percentage of individuals in each occupational category who had completed at least one year of college, based on 1950 census data. It reflects the average educational attainment of workers in that occupation. Occupational Prestige Score: This score is based on public perceptions of social standing for each occupation, derived from 1960 s NORC surveys and assigned using the 1950 occupational classification. It captures the relative societal prestige attributed to various occupations.
