# Supplementary material for: The effects of parental education on male mortality: evidence from the first wave of compulsory schooling laws
Source: J Popul Econ. 2025 Nov 12;38(4):77. doi: 10.1007/s00148-025-01134-y (PMC12605591; doi:10.1007/s00148-025-01134-y)
Supplement: Supplementary file 1 — (PDF 1.01 MB) [file 148_2025_1134_MOESM1_ESM.pdf]

Appendix A

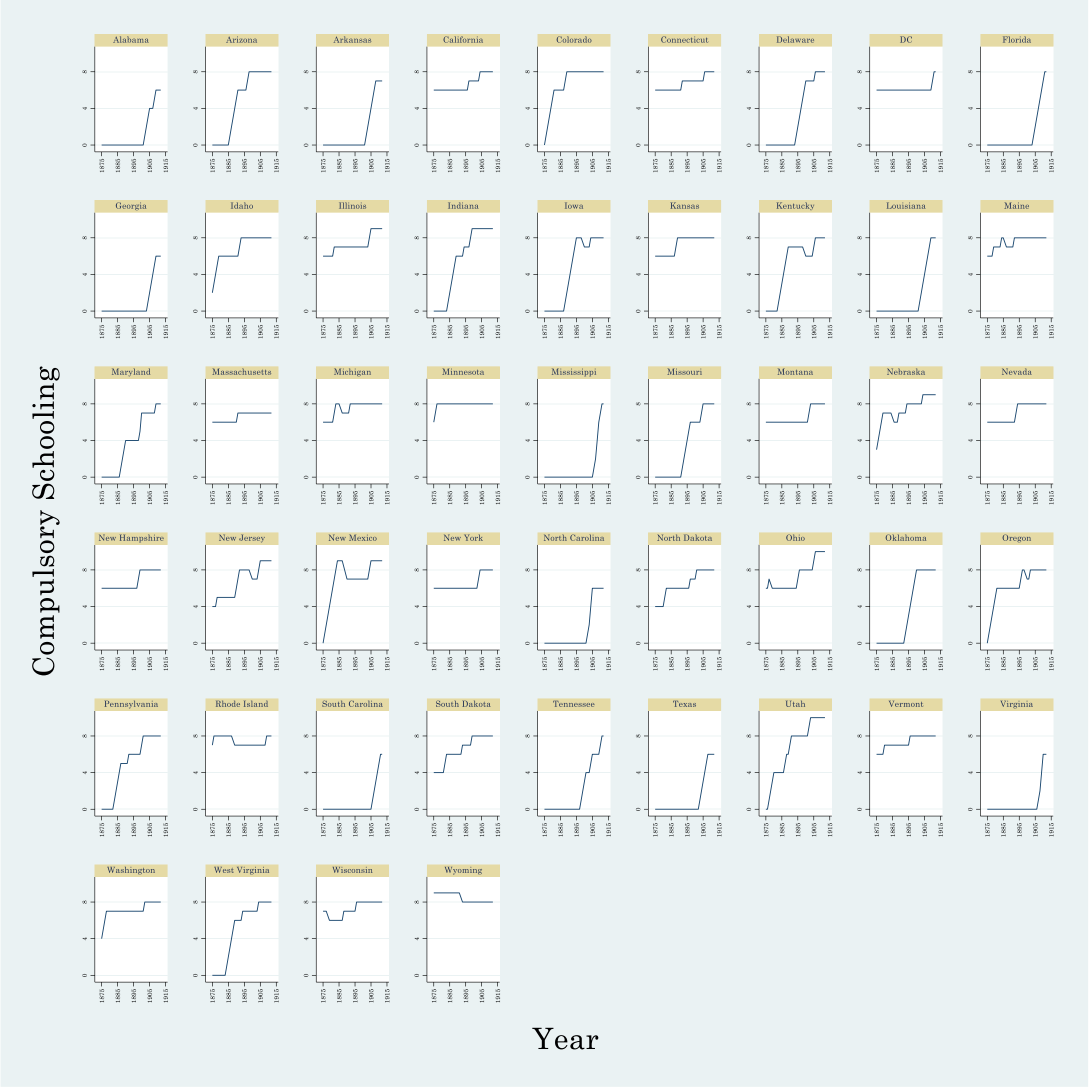

Appendix Figure A-1 - Compulsory Schooling Laws Across States and Time

Appendix Table A-1 - Comparing Statistics of Original Population and Final Sample

	Original Population				Final Sample			
	**Mean**	**SD**	**Min**	**Max**	**Mean**	**SD**	**Min**	**Max**
Birth Year	1929.157	6.80264	1900	1940	1925.7792	6.70995	1900	1940
Father's Birth Year	1898.3012	7.9782	1882	1912	1896.3643	7.81665	1882	1915
Father's years of schooling	8.071	3.53562	0	20	7.9934	3.32794	0	23
Years of schooling	6.50565	3.81015	0	20	7.06715	3.87754	0	20
Family Socioeconomic Index	11.90566	18.76466	0	96	22.52312	17.2667	3	96
Nonwhite	.10648	.30845	0	1	.06606	.24839	0	1
Hispanic	.01269	.11195	0	1	.01107	.10465	0	1
Father's Birth-State Compulsory schooling 1-5 Years	.11073	.31379	0	1	.11046	.31347	0	1
Father's Birth-State Compulsory schooling 6 Years	.17987	.38408	0	1	.25093	.43355	0	1
Father's Birth-State Compulsory schooling 7 Years	.15445	.36138	0	1	.15596	.36282	0	1
Father's Birth-State Compulsory schooling 8 Years	.26017	.43873	0	1	.12328	.32876	0	1
Father's Birth-State Compulsory schooling 9-10 Years	.05436	.22673	0	1	.00929	.09592	0	1
Observations	30,002,097				1,925,362			

Appendix Table A-2 - Summary Statistics of the Sample across States with Different Educational Policies

	State-Level Schooling Policies									
	1-5 Years		6 Years		7 Years		8 Years		9-10 Years	
	Mean	SD	Mean	SD	Mean	SD	Mean	SD	Mean	SD
Death Age (Months)	818.13729	117.85625	828.07543	117.12139	803.08922	116.09978	779.24145	115.41362	744.64254	111.95068
Birth Year	1925.4065	6.49454	1924.532	6.55174	1927.1377	6.21901	1929.6331	6.07036	1933.0246	5.01814
Death Year	1993.5874	8.41884	1993.54	8.42205	1994.0645	8.39134	1994.5685	8.31126	1995.0775	8.4
White	.95212	.21352	.98907	.10398	.98962	.10134	.99085	.09521	.9884	.10709
Black	.04539	.20817	.00765	.08711	.00798	.08897	.00531	.07268	.00743	.0859
Hispanic	.01019	.10042	.00629	.07906	.01149	.10658	.00801	.08916	.06071	.23881
Father's Birth Year	1896.5735	7.07681	1894.7078	7.08346	1899.0497	6.45531	1902.891	7.14554	1908.6531	6.32789
Mother's Birth Year	1899.7483	7.20175	1898.1661	7.31654	1902.0164	6.75373	1905.2908	6.94501	1909.9956	5.55013
Father's Years Schooling	7.95423	3.20168	8.69113	3.02304	8.601	3.00991	9.05992	2.82973	9.4207	2.92288
Mother's Years Schooling	8.27713	2.85783	8.8203	2.67466	8.85383	2.69292	9.38569	2.5753	9.63497	2.73657
Observation	211435		480559		298652		235970		17756	

Appendix Table A-3 - State-Level Educational Policies and Successful Data Linking from 1940-Census and Death Records

	Outcome: Successful Merging between 1940-Census and Death Records	
	Father Sample	Mother Sample
	(1)	(2)
Law Requires 1-5 Years	-.00027	.00052*
	(.0006)	(.00027)
Law Requires 6 Years	-.0016	-.0001
	(.00101)	(.00036)
Law Requires 7 Years	-.00123	-.00025
	(.00098)	(.00043)
Law Requires 8 Years	-.00247**	-.00031
	(.00112)	(.00044)
Law Requires 9-10 Years	-.00229	-.00009
	(.00154)	(.00055)
Mean DV	0.058	0.049
Observations	31797923	34851073

Notes. Standard errors, clustered on father's birth-state-birth-year are in parentheses. Regressions include child's birth-county fixed effects, child's birth year fixed effects, parental birth-state fixed effects, and parental region-of-birth-by-birth-cohort fixed effects. Regressions also include race and ethnicity dummies as individual covariates.
*** p<0.01, ** p<0.05, * p<0.1

Appendix Table A-4 - Effect of Mothers' Education on Sons' Longevity

	Outcome: Age-at-Death (Months)/Sample/Method:					
	Full		Whites		Nonwhites	
	OLS	IV	OLS	IV	OLS	IV
	(1)	(2)	(3)	(4)	(5)	(6)
Father's Years of Schooling	.76818***	9.91875***	.81349***	8.67247***	.29076***	14.97119***
	(.02924)	(3.20347)	(.03077)	(3.36538)	(.10238)	(5.75783)
Mean DV	813.831	813.831	815.692	815.692	788.193	788.252
Observations	1835832	1835834	1711306	1711309	124272	124525
First-Stage F-Statistic		11.639		11.200		7.447

Notes. Standard errors, clustered on father's birth-state-birth-year are in parentheses. Regressions include child's birth-county fixed effects, child's birth year fixed effects, parental birth-state fixed effects, and parental region-of-birth-by-birth-cohort fixed effects. Regressions also include race and ethnicity dummies as individual covariates.

*** p<0.01, ** p<0.05, * p<0.1

Appendix Table A-5 - Additional Heterogeneity in the Effects of Fathers' education on Son's Longevity

	Outcome: Age-at-Death (Months)/Sample:							
	Father's Years of Schooling < 12	Father's Years of Schooling ≥ 12	Father's Socioeconomic Index < Median	Father's Socioeconomic Index ≥ Median	Father's Birth Region Northeast	Father's Birth Region Midwest	Father's Birth Region South	Father's Birth Region West
	(1)	(2)	(3)	(4)	(5)	(6)	(7)	(8)
Father's Years of Schooling	8.62105**	3.61926	4.35102	6.73639*	1.19278	2.1355	3.24618	9.16344
	(4.04209)	(9.80419)	(3.55429)	(3.87234)	(2.20687)	(3.94322)	(2.46187)	(13.12404)
Observations	1615451	309911	968120	957242	428508	698863	653835	144131
R-Squared	.27019	.31884	.2907	.26576	.29842	.29374	.27996	.26693
Mean DV	814.480	812.154	811.356	816.887	815.884	819.702	807.196	813.034
First-Stage F-Statistic	12.157	5.344	12.334	9.052	19.270	9.309	17.275	0.814

Notes. Standard errors, clustered on father's birth-state-birth-year are in parentheses. Regressions include child's birth-county fixed effects, child's birth year fixed effects, parental birth-state fixed effects, and parental region-of-birth-by-birth-cohort fixed effects. Regressions also include race and ethnicity dummies as individual covariates.

*** p<0.01, ** p<0.05, * p<0.1

Appendix Table A-6 - State-Level Educational Policies and Sample's Racial Composition Change

	Outcomes:		
	White	Black	Hispanic
	(1)	(2)	(3)
Law Requires 1-5 Years	.00796***	-.00636***	-.00022
	(.00222)	(.00211)	(.0006)
Law Requires 6 Years	.01034***	-.00872***	-.00225***
	(.00223)	(.00213)	(.00068)
Law Requires 7 Years	.01509***	-.01308***	-.00199***
	(.00285)	(.00277)	(.00077)
Law Requires 8 Years	.01427***	-.01152***	-.00376***
	(.00298)	(.00278)	(.00111)
Law Requires 9-10 Years	.01547***	-.01378***	-.00117
	(.00373)	(.00348)	(.00294)
Mean DV	0.934	0.063	0.011
Observations	1906959	1906959	1906959

Notes. Standard errors, clustered on father's birth-state-birth-year are in parentheses. Regressions include child's birth-county fixed effects, child's birth year fixed effects, parental birth-state fixed effects, and parental region-of-birth-by-birth-cohort fixed effects. Regressions also include race and ethnicity dummies as individual covariates.
*** p<0.01, ** p<0.05, * p<0.1

Appendix Table A-7 - Effect of Compulsory Schooling Laws on Father's Schooling, by Race

	Outcome: Years of Schooling, Sample:		
	Full	Whites	Non-Whites
	(1)	(2)	(3)
Law Requires 1-5 Years	.00946	-.0209	.19958***
	(.01815)	(.01646)	(.05776)
Law Requires 6 Years	.0963***	.04947***	.2697***
	(.01988)	(.01863)	(.07902)
Law Requires 7 Years	.08689***	.02542	.40395***
	(.02275)	(.02087)	(.09408)
Law Requires 8 Years	.20907***	.14208***	.50399***
	(.02838)	(.02632)	(.12337)
Law Requires 9-10 Years	.1856***	.12059***	1.12594***
	(.03903)	(.03824)	(.28333)
Mean DV	7.997	8.212	4.929
Observations	1913592	1787864	125474

Notes. Standard errors, clustered on father's birth-state-birth-year are in parentheses. Regressions include child's birth-county fixed effects, child's birth year fixed effects, parental birth-state fixed effects, and parental region-of-birth-by-birth-cohort fixed effects. Regressions also include race and ethnicity dummies as individual covariates. *** p<0.01, ** p<0.05, * p<0.1

Appendix Table A-8 - State-Level Educational Policies and State-Level Characteristics

	Outcomes:						
	Share of Dry Counties	Birth Registration Law Effective in State	Suffrage Law Effective in State	Poll Tax Effective in State	Socioeconomic Index	Male Labor Force Participation Rate	Share of Literate
	(1)	(2)	(3)	(4)	(5)	(6)	(7)
Law Requires 1-5 Years	.03186	-.00214	-.00392	.00845	-.15064***	.00093	.00061
	(.0353)	(.00497)	(.00468)	(.02631)	(.03909)	(.00058)	(.0015)
Law Requires 6 Years	.05204	-.0085	-.01818*	-.02295	-.20405***	.00094	-.00459***
	(.03615)	(.00644)	(.01036)	(.02666)	(.03873)	(.00063)	(.00156)
Law Requires 7 Years	.02856	-.00959	-.0393**	-.15632***	-.13995***	.0014*	-.01027***
	(.04227)	(.00894)	(.01969)	(.02929)	(.04645)	(.00083)	(.00208)
Law Requires 8 Years	-.03236	-.01546	.00932	-.07975**	-.17551***	.00177*	-.01326***
	(.04015)	(.01457)	(.0153)	(.03215)	(.05018)	(.00093)	(.00217)
Law Requires 9-10 Years	-.04542	-.10033*	.04443	-.08751***	-.18182**	.01051***	-.01364***
	(.05223)	(.05145)	(.06099)	(.03239)	(.07936)	(.00176)	(.00254)
Mean DV	0.149	0.012	0.027	0.254	22.663	0.520	0.907
Observations	1603	1603	1637	1603	1637	1634	1637

Notes. Standard errors, clustered on state-year are in parentheses. Regressions include state fixed effects and region-by-year fixed effects.
*** p<0.01, ** p<0.05, * p<0.1

Appendix Table A-9 - Effect of Mothers' Education on Sons' Education and Socioeconomic Status (SES)

	Outcome/Method:					
	Schooling/(Age-6) Conditional on Age ≥ 6	Schooling/(Age-6) Conditional on Age ≥ 6	Schooling Conditional on Age ≥ 16	Schooling Conditional on Age ≥ 16	SES Score Conditional on Age > 17	SES Score Conditional on Age > 17
	OLS	IV	OLS	IV	OLS	IV
	(1)	(2)	(3)	(4)	(5)	(6)
Father's Years of	.09159***	.18709***	.40789***	1.15304***	1.48679***	4.6143**
Schooling	(.00208)	(.06285)	(.0045)	(.26088)	(.01439)	(1.95)
Mean DV	2.140	2.140	9.983	9.983	22.573	22.573
Observations	1491332	1491332	699177	699177	428729	428729
First-Stage F-Statistic		12.509		2.756		1.737

Notes. Standard errors, clustered on father's birth-state-birth-year are in parentheses. Regressions include child's birth-county fixed effects, child's birth year fixed effects, parental birth-state fixed effects, and parental region-of-birth-by-birth-cohort fixed effects. Regressions also include race and ethnicity dummies as individual covariates.

*** p<0.01, ** p<0.05, * p<0.1

Appendix Table A-10 - Parental Education and Age-at-First-Birth of the Child

	Outcome: Age at Birth of the First Child			
	OLS	IV	OLS	IV
	(1)	(2)	(3)	(4)
Father's Years of Schooling	.11611***	-.62399***		
	(.00218)	(.15254)		
Mother's Years of Schooling			.21136***	-.7407**
			(.00252)	(.34539)
Mean DV	26.989	26.989		
Observations	1913592	1913592	1835832	1835832
First-Stage F-Stata		16.522		9.530

Notes. Standard errors, clustered on father's birth-state-birth-year are in parentheses. Regressions include child's birth-county fixed effects, child's birth year fixed effects, parental birth-state fixed effects, and parental region-of-birth-by-birth-cohort fixed effects. Regressions also include race and ethnicity dummies as individual covariates.
*** p<0.01, ** p<0.05, * p<0.1

Appendix Table A-11 - Robustness Check of Father's Schooling on Longevity: Adding Birth-State FE by Race/Ethnicity Dummies and Birth-Year FE by Race/Ethnicity Dummies

	Outcome: Age-at-Death (Months)/Sample/Method:					
	Full		Whites		Nonwhites	
	OLS	IV	OLS	IV	OLS	IV
	(1)	(2)	(3)	(4)	(5)	(6)
Father's Years of	.70728***	5.27593**	.7191***	4.85267*	.61835***	7.82871
Schooling	(.02533)	(2.55891)	(.0259)	(2.60855)	(.10253)	(5.43456)
Mean DV	814.190	814.190	816.006	816.006	788.323	788.364
Observations	1913585	1913593	1787858	1787866	125466	125727
First-Stage F-Statistic		15.386		15.354		6.476

Notes. Standard errors, clustered on father's birth-state-birth-year are in parentheses. Regressions include child's birth-county fixed effects, child's birth year fixed effects, parental birth-state fixed effects, and parental region-of-birth-by-birth-cohort fixed effects. Regressions also include race and ethnicity dummies as individual covariates.
*** p<0.01, ** p<0.05, * p<0.1

Appendix Table A-12 - Robustness Check of Father's Schooling on Longevity: Adding State Controls

	Outcome: Age-at-Death (Months)/Sample/Method:					
	Full		Whites		Nonwhites	
	OLS	IV	OLS	IV	OLS	IV
	(1)	(2)	(3)	(4)	(5)	(6)
Father's Years of	.6627***	5.76375*	.67909***	4.71133	.51891***	14.63638
Schooling	(.02611)	(3.46177)	(.02662)	(3.4665)	(.10396)	(13.41769)
Mean DV	813.794	813.794	815.662	815.661	788.096	788.149
Observations	1834624	1834626	1710128	1710131	124246	124495
First-Stage F-Statistic		11.747		11.795		1.709

Notes. Standard errors, clustered on father's birth-state-birth-year are in parentheses. Regressions include child's birth-county fixed effects, child's birth year fixed effects, parental birth-state fixed effects, and parental region-of-birth-by-birth-cohort fixed effects. Regressions also include race and ethnicity dummies as individual covariates.

*** p<0.01, ** p<0.05, * p<0.1

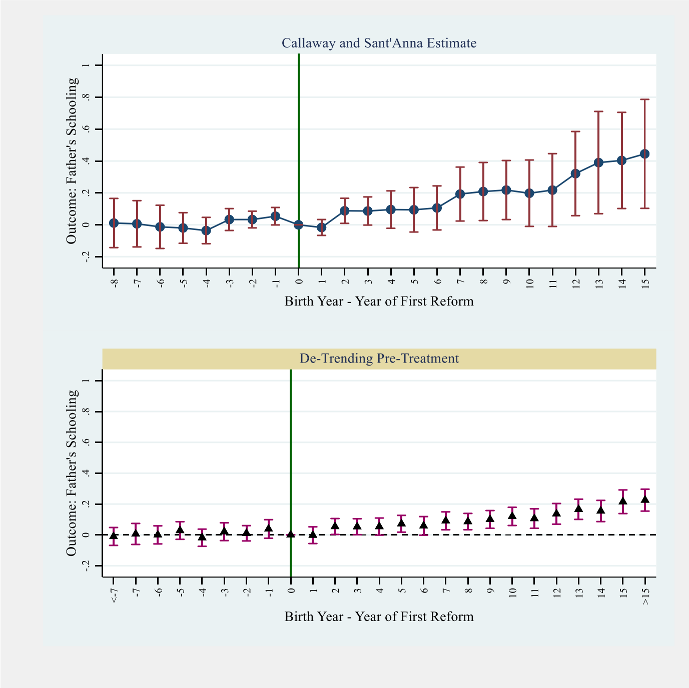

Appendix Figure A-1 - Event Study Analysis of Compulsory Schooling Laws, Robustness to Alternative Models

Notes. Regressions include child's birth-county fixed effects, child's birth year fixed effects, parental birth-state fixed effects, and parental region-of-birth-by-birth-cohort fixed effects. Regressions also include race and ethnicity dummies as individual covariates.

Appendix Table A-13 - Replicating the Main Results Using Numident Data Covering Death Years 1988 – 2005

	Outcome: Age-at-Death (Months)/Sample/Method:							
	Males		Females		Males		Females	
	OLS	IV	OLS	IV	OLS	IV	OLS	IV
	(1)	(2)	(3)	(4)	(5)	(6)	(7)	(8)
Father's Years of Schooling	.44784***	-1.37927	.24391***	-.26895				
	(.01246)	(1.07712)	(.01501)	(1.41743)				
Mother's Years of Schooling					.51739***	1.17554	.24352***	.13297
					(.01499)	(1.37974)	(.01712)	(2.03895)
Observations	2382836	2382836	1648927	1648930	2192495	2192504	1542324	1542339
R-Squared	.60108	.59719	.6047	.6044	.58136	.58096	.60386	.60385
Mean DV	862.303	862.303	868.147	868.147	857.063	857.063	866.399	866.399
First-Stage F-Statistic		19.115		17.839		11.468		6.834

Notes. Standard errors, clustered on father's birth-state-birth-year are in parentheses. Regressions include child's birth-county fixed effects, child's birth year fixed effects, parental birth-state fixed effects, and parental region-of-birth-by-birth-cohort fixed effects. Regressions also include race and ethnicity dummies as individual covariates.
*** p<0.01, ** p<0.05, * p<0.1

Appendix Table A-14 - Adding Father's Birth State Trend

	Outcome: Age at Death (Months)	
	OLS	IV
	(1)	(2)
Father's Years of Schooling	.6549***	6.50221
	(.0252)	(5.26674)
Mean DV	814.106	814.106
Observations	1925361	1925362
First-Stage F-Statistic		5.464

Notes. Standard errors, clustered on father's birth-state-birth-year are in parentheses. Regressions include child's birth-county fixed effects, child's birth year fixed effects, parental birth-state fixed effects, and parental region-of-birth-by-birth-cohort fixed effects. Regressions also include race and ethnicity dummies as individual covariates.
*** p<0.01, ** p<0.05, * p<0.1

Appendix Table A-15 - Robustness of the Results to Alternative Controls

	Outcome: Age-at-Death (Months):										
	Main Results	1890 State-Level Infant Mortality Rate Interacted with Parental Birth Cohort FE	1918 – 1919 Influenza Pandemic Control	1916 Polio Pandemic Control	Exposure to Malaria Interacted with Parental Birth Cohort FE	Exposure to Hookworm Interacted with Parental Birth Cohort FE	Average School Attendance Rate Control	Average Public School Year Length Control	Average Term Length Control	Parental Exposure to Prohibition	Parental Exposure to Birth Registration Laws
	(1)	(2)	(3)	(4)	(5)	(6)	(7)	(8)	(9)	(10)	(11)
Father's Years of Schooling	5.82148**	8.441***	7.6085***	5.49032**	6.12217**	6.64091**	9.12269*	8.03736*	8.05951*	5.82148**	5.81854**
	(2.5471)	(3.2455)	(2.40736)	(2.49872)	(2.82501)	(2.89677)	(4.89126)	(4.71617)	(4.74379)	(2.54711)	(2.54894)
Mean DV	1925361	1925361	1765314	1778533	1925361	1902776	757524	757524	757524	1925361	1923050
Observations	814.106	814.106	814.257	815.136	814.106	814.405	771.175	771.175	771.175	814.106	814.106
First-Stage F-Statistic	14.924	11.681	20.945	17.043	13.610	12.536	10.151	11.315	11.311	14.924	

Notes. Standard errors, clustered on father's birth-state-birth-year are in parentheses. Regressions include child's birth-county fixed effects, child's birth year fixed effects, parental birth-state fixed effects, and parental region-of-birth-by-birth-cohort fixed effects. Regressions also include race and ethnicity dummies as individual covariates.

In column 2, the 1890 state level infant mortality rate is extracted from (Bleakley, 2010). In column 3, state-level prevalence of influenza is extracted from the Tycho project (Tycho, 2021) and interacted with parental birth cohort fixed effects. In column 4, the 1916 polio prevalence is extracted from the Tycho project (Tycho, 2021) and interacted with parental birth cohort fixed effects. In column 5, state level malaria risk index is extracted from (Bleakley, 2010) and interacted with parental birth cohort fixed effects. In column 5, hookworm rate for 1910 is extracted from (Bleakley, 2010) and interacted with parental birth cohort fixed effects.

*** p<0.01, ** p<0.05, * p<0.1

Appendix Table A-16 - Employing Heckman (1979) Two-Step Model

	Outcome: Age at Death (Months)	
	OLS	IV
	(1)	(2)
Father's Years of Schooling	.65367***	5.82148**
	(.02521)	(2.5471)
Mean DV	814.106	814.106
Observations	1925361	1925361
First-Stage F-Statistic		14.924
First-Step Regression Observations	31,797,923	

Notes. Standard errors, clustered on father's birth-state-birth-year are in parentheses. Regressions include child's birth-county fixed effects, child's birth year fixed effects, parental birth-state fixed effects, and parental region-of-birth-by-birth-cohort fixed effects. Regressions also include race and ethnicity dummies as individual covariates.
*** p<0.01, ** p<0.05, * p<0.1

Appendix Table A-17 - Exploring Nonlinearity in the Effects Using Alternative Measures of Fathers' education

	Outcomes: Age-at-Death (Months)								
	(1)	(2)	(3)	(4)	(5)	(6)	(7)	(8)	(9)
Father's Years of Schooling > 4	73.63182***								
	(28.12512)								
Father's Years of Schooling > 5		40.11789***							
		(15.42231)							
Father's Years of Schooling > 6			37.37707***						
			(14.02259)						
Father's Years of Schooling > 7				34.91329**					
				(14.81561)					
Father's Years of Schooling > 8					9.18081				
					(12.25532)				
Father's Years of Schooling > 9						7.21017			
						(16.08608)			
Father's Years of Schooling > 10							19.21615		
							(19.80803)		
Father's Years of Schooling > 11								45.67461*	
								(26.17893)	
Father's Years of Schooling > 12									68.17006
									(101.28364)
Observations	1925362	1925362	1925362	1925362	1925362	1925362	1925362	1925362	1925362
Mean DV	814.106	814.106	814.106	814.106	814.106	814.106	814.106	814.106	814.106
First-Stage F-Stat	9.902	20.636	19.193	15.218	27.454	20.515	17.431	13.260	1.731

Notes. Standard errors, clustered on father's birth-state-birth-year are in parentheses. Regressions include child's birth-county fixed effects, child's birth year fixed effects, parental birth-state fixed effects, and parental region-of-birth-by-birth-cohort fixed effects. Regressions also include race and ethnicity dummies as individual covariates.
*** p<0.01, ** p<0.05, * p<0.1

Appendix Table A-18 - Effect of Compulsory Schooling Laws on Mother's Education

	Outcomes:		
	Years of Schooling	Years of Schooling>4	Years of Schooling>7
	(1)	(2)	(3)
Law Requires 1-5 Years	-.00589	-.00031	-.00008
	(.01762)	(.00166)	(.00296)
Law Requires 6 Years	.07021***	.00639***	.00824***
	(.01937)	(.00176)	(.00317)
Law Requires 7 Years	.08571***	.00395**	.00683*
	(.02189)	(.00193)	(.0037)
Law Requires 8 Years	.16375***	.00503**	.02014***
	(.02501)	(.00225)	(.00431)
Law Requires 9-10 Years	.14569***	-.00342	.01108**
	(.03165)	(.00326)	(.00494)
Mean DV	8.383	0.913	0.680
Observations	1835832	1843513	1843513

Notes. Standard errors, clustered on father's birth-state-birth-year are in parentheses. Regressions include child's birth-county fixed effects, child's birth year fixed effects, parental birth-state fixed effects, and parental region-of-birth-by-birth-cohort fixed effects. Regressions also include race and ethnicity dummies as individual covariates. *** p<0.01, ** p<0.05, * p<0.1

Appendix Table A-19 - Exploring Mechanisms Using Information in the 1940 Census

	Outcomes:				
	Average Schooling in the County of Residence	Occupational Income Score	Occupational Earnings Score	Occupational Education Score	Occupational Prestige Score
	(1)	(2)	(3)	(4)	(5)
Father's Years of	.01049	1.90596***	5.9763***	1.22914	1.38273*
Schooling	(.00722)	(.73879)	(2.16078)	(1.00353)	(.83562)
Mean DV	6.191	18.822	35.103	8.848	27.889
Observations	1925351	460635	459993	458344	459993
First-Stage F-Stata	32.965	4.113	4.026	3.942	4.026

Notes. Standard errors, clustered on father's birth-state-birth-year are in parentheses. Regressions include child's birth-county fixed effects, child's birth year fixed effects, parental birth-state fixed effects, and parental region-of-birth-by-birth-cohort fixed effects. Regressions also include race and ethnicity dummies as individual covariates.
*** p<0.01, ** p<0.05, * p<0.1
